# Supplementary material for: Staphylococcus aureus Pathogenicity in Cystic Fibrosis Patients—Results from an Observational Prospective Multicenter Study Concerning Virulence Genes, Phylogeny, and Gene Plasticity
Source: Toxins (Basel). 2020 Apr 26;12(5):279. doi: 10.3390/toxins12050279 (PMC7290773; doi:10.3390/toxins12050279)
Supplement: Supplementary file 1 [file toxins-12-00279-s001.pdf]

Supplementary Materials: *Staphylococcus aureus* Pathogenicity in Cystic Fibrosis Patients—Results from an Observational Prospective Multicenter Study Concerning Virulence Genes, Phylogeny, and Gene Plasticity

Jonas Lange, Kathrin Heidenreich, Katharina Higelin, Kristina Dyck, Vanessa Marx, Christian Reichel, Willem van Wamel, Martijn den Reijer, Dennis Goerlich and Barbara C. Kahl

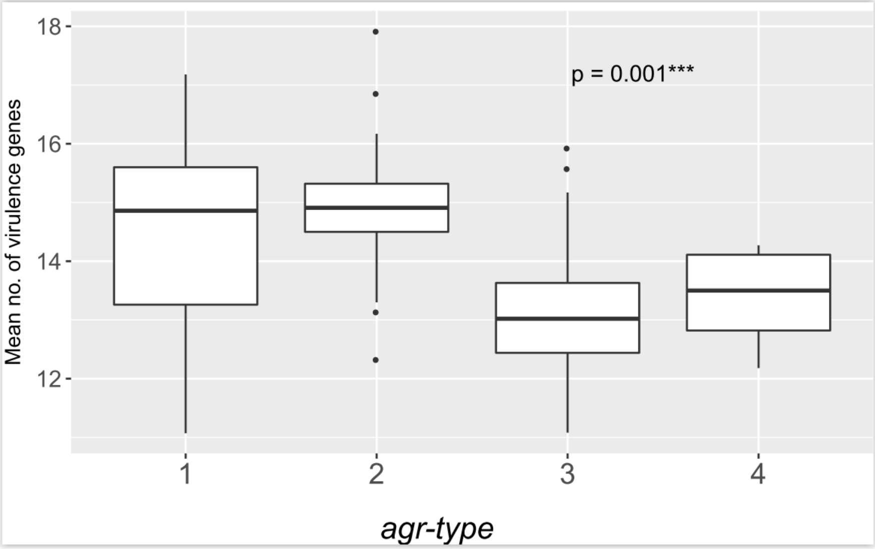

Figure S1. *Agr*-type vs. virulence gene count.

Table S1. *Spa*-types detected among the 3180 *S. aureus* isolates.

Number of *spa*-Types	*Spa*-Type	Frequency	Percent	Valid Percent	Cumulative Percent
	n.t.	6	0.2	0.2	0.2
**1**	t002	85	2.7	2.7	2.9
**2**	t003	21	0.7	0.7	3.5
**3**	t004	13	0.4	0.4	3.9
**4**	t005	29	0.9	0.9	4.8
**5**	t006	12	0.4	0.4	5.2
**6**	t008	117	3.7	3.7	8.9
**7**	t009	8	0.3	0.3	9.2
**8**	t010	2	0.1	0.1	9.2
**9**	t011	11	0.3	0.3	9.6
**10**	t012	149	4.7	4.7	14.2
**11**	t015	100	3.1	3.1	17.4
**12**	t017	15	0.5	0.5	17.9
**13**	t018	41	1.3	1.3	19.2
**14**	t019	21	0.7	0.7	19.8
**15**	t021	48	1.5	1.5	21.3
**16**	t022	13	0.4	0.4	21.7
**17**	t024	26	0.8	0.8	22.5

**18**	t026	61	1.9	1.9	24.5
**19**	t034	21	0.7	0.7	25.1
**20**	t040	4	0.1	0.1	25.3
**21**	t044	12	0.4	0.4	25.6
**22**	t045	5	0.2	0.2	25.8
**23**	t050	51	1.6	1.6	27.4
**24**	t056	60	1.9	1.9	29.3
**25**	t065	44	1.4	1.4	30.7
**26**	t068	1	0.0	0.0	30.7
**27**	t073	11	0.3	0.3	31.0
**28**	t076	1	0.0	0.0	31.1
**29**	t078	62	1.9	1.9	33.0
**30**	t081	22	0.7	0.7	33.7
**31**	t084	241	7.6	7.6	41.3
**32**	t085	11	0.3	0.3	41.6
**33**	t091	137	4.3	4.3	45.9
**34**	t094	4	0.1	0.1	46.1
**35**	t099	1	0.0	0.0	46.1
**36**	t100	8	0.3	0.3	46.4
**37**	t103	1	0.0	0.0	46.4
**38**	t105	4	0.1	0.1	46.5
**39**	t1050	19	0.6	0.6	47.1
**40**	t1057	2	0.1	0.1	47.2
**41**	t10605	6	0.2	0.2	47.4
**42**	t10606	2	0.1	0.1	47.4
**43**	t1070	2	0.1	0.1	47.5
**44**	t108	3	0.1	0.1	47.6
**45**	t116	18	0.6	0.6	48.1
**46**	t1201	1	0.0	0.0	48.2
**47**	t1203	3	0.1	0.1	48.3
**48**	t1211	24	0.8	0.8	49.0
**49**	t122	36	1.1	1.1	50.2
**50**	t1245	2	0.1	0.1	50.2
**51**	t1259	2	0.1	0.1	50.3
**52**	t12674	7	0.2	0.2	50.5
**53**	t12678	1	0.0	0.0	50.5
**54**	t12679	4	0.1	0.1	50.7
**55**	t12680	2	0.1	0.1	50.7
**56**	t12681	1	0.0	0.0	50.8
**57**	t127	37	1.2	1.2	51.9
**58**	t129	11	0.3	0.3	52.3
**59**	t1333	4	0.1	0.1	52.4
**60**	t1345	1	0.0	0.0	52.4
**61**	t136	8	0.3	0.3	52.7
**62**	t138	1	0.0	0.0	52.7
**63**	t1406	2	0.1	0.1	52.8
**64**	t1416	2	0.1	0.1	52.8
**65**	t144	7	0.2	0.2	53.1
**66**	t1451	28	0.9	0.9	53.9

**67**	t1491	1	0.0	0.0	54.0
**68**	t1492	2	0.1	0.1	54.0
**69**	t150	4	0.1	0.1	54.2
**70**	t1510	14	0.4	0.4	54.6
**71**	t153	14	0.4	0.4	55.0
**72**	t1541	1	0.0	0.0	55.1
**73**	t1544	1	0.0	0.0	55.1
**74**	t156	6	0.2	0.2	55.3
**75**	t1574	1	0.0	0.0	55.3
**76**	t1577	23	0.7	0.7	56.0
**77**	t159	33	1.0	1.0	57.1
**78**	t162	1	0.0	0.0	57.1
**79**	t164	5	0.2	0.2	57.3
**80**	t1652	8	0.3	0.3	57.5
**81**	t166	38	1.2	1.2	58.7
**82**	t1670	9	0.3	0.3	59.0
**83**	t1671	6	0.2	0.2	59.2
**84**	t1685	2	0.1	0.1	59.2
**85**	t1689	2	0.1	0.1	59.3
**86**	t169	1	0.0	0.0	59.3
**87**	t1707	7	0.2	0.2	59.6
**88**	t1709	1	0.0	0.0	59.6
**89**	t179	12	0.4	0.4	60.0
**90**	t185	17	0.5	0.5	60.5
**91**	t1858	2	0.1	0.1	60.6
**92**	t186	4	0.1	0.1	60.7
**93**	t189	11	0.3	0.3	61.0
**94**	t190	17	0.5	0.5	61.6
**95**	t193	2	0.1	0.1	61.6
**96**	t1978	13	0.4	0.4	62.0
**97**	t1991	20	0.6	0.6	62.7
**98**	t2065	3	0.1	0.1	62.8
**99**	t2080	21	0.7	0.7	63.4
**100**	t209	42	1.3	1.3	64.7
**101**	t211	16	0.5	0.5	65.3
**102**	t2133	2	0.1	0.1	65.3
**103**	t2164	5	0.2	0.2	65.5
**104**	t223	2	0.1	0.1	65.5
**105**	t227	1	0.0	0.0	65.6
**106**	t2275	1	0.0	0.0	65.6
**107**	t228	1	0.0	0.0	65.6
**108**	t230	2	0.1	0.1	65.7
**109**	t2309	1	0.0	0.0	65.7
**110**	t2351	1	0.0	0.0	65.8
**111**	t2375	1	0.0	0.0	65.8
**112**	t2383	20	0.6	0.6	66.4
**113**	t2398	4	0.1	0.1	66.5
**114**	t240	10	0.3	0.3	66.9
**115**	t2419	3	0.1	0.1	66.9

**116**	t2439	21	0.7	0.7	67.6
**117**	t2441	24	0.8	0.8	68.4
**118**	t246	2	0.1	0.1	68.4
**119**	t2509	17	0.5	0.5	69.0
**120**	t254	16	0.5	0.5	69.5
**121**	t2553	3	0.1	0.1	69.6
**122**	t258	11	0.3	0.3	69.9
**123**	t2666	8	0.3	0.3	70.2
**124**	t272	11	0.3	0.3	70.5
**125**	t275	5	0.2	0.2	70.7
**126**	t277	1	0.0	0.0	70.7
**127**	t2787	14	0.4	0.4	71.1
**128**	t279	16	0.5	0.5	71.6
**129**	t2802	16	0.5	0.5	72.1
**130**	t284	10	0.3	0.3	72.5
**131**	t2845	12	0.4	0.4	72.8
**132**	t289	1	0.0	0.0	72.9
**133**	t2919	4	0.1	0.1	73.0
**134**	t2949	1	0.0	0.0	73.0
**135**	t295	1	0.0	0.0	73.1
**136**	t3012	1	0.0	0.0	73.1
**137**	t304	4	0.1	0.1	73.2
**138**	t306	23	0.7	0.7	73.9
**139**	t308	3	0.1	0.1	74.0
**140**	t3258	1	0.0	0.0	74.1
**141**	t330	8	0.3	0.3	74.3
**142**	t331	18	0.6	0.6	74.9
**143**	t3331	2	0.1	0.1	74.9
**144**	t338	16	0.5	0.5	75.4
**145**	t346	56	1.8	1.8	77.2
**146**	t352	1	0.0	0.0	77.2
**147**	t359	12	0.4	0.4	77.6
**148**	t362	21	0.7	0.7	78.3
**149**	t363	1	0.0	0.0	78.3
**150**	t364	3	0.1	0.1	78.4
**151**	t3667	1	0.0	0.0	78.4
**152**	t370	2	0.1	0.1	78.5
**153**	t3745	14	0.4	0.4	78.9
**154**	t377	1	0.0	0.0	79.0
**155**	t390	2	0.1	0.1	79.0
**156**	t393	3	0.1	0.1	79.1
**157**	t3933	6	0.2	0.2	79.3
**158**	t394	7	0.2	0.2	79.5
**159**	t4069	1	0.0	0.0	79.6
**160**	t4096	1	0.0	0.0	79.6
**161**	t4228	2	0.1	0.1	79.7
**162**	t4325	17	0.5	0.5	80.2
**163**	t435	2	0.1	0.1	80.3
**164**	t4451	8	0.3	0.3	80.5

**165**	t447	4	0.1	0.1	80.6
**166**	t449	10	0.3	0.3	80.9
**167**	t4570	1	0.0	0.0	81.0
**168**	t466	1	0.0	0.0	81.0
**169**	t4870	1	0.0	0.0	81.0
**170**	t488	1	0.0	0.0	81.1
**171**	t491	6	0.2	0.2	81.3
**172**	t493	2	0.1	0.1	81.3
**173**	t4989	31	1.0	1.0	82.3
**174**	t499	14	0.4	0.4	82.7
**175**	t505	16	0.5	0.5	83.2
**176**	t5088	5	0.2	0.2	83.4
**177**	t509	6	0.2	0.2	83.6
**178**	t514	16	0.5	0.5	84.1
**179**	t5152	8	0.3	0.3	84.3
**180**	t5154	2	0.1	0.1	84.4
**181**	t521	3	0.1	0.1	84.5
**182**	t5210	2	0.1	0.1	84.6
**183**	t524	3	0.1	0.1	84.7
**184**	t5306	1	0.0	0.0	84.7
**185**	t539	8	0.3	0.3	84.9
**186**	t5430	13	0.4	0.4	85.3
**187**	t548	46	1.4	1.4	86.8
**188**	t550	4	0.1	0.1	86.9
**189**	t5520	1	0.0	0.0	86.9
**190**	t559	9	0.3	0.3	87.2
**191**	t5682	6	0.2	0.2	87.4
**192**	t5683	10	0.3	0.3	87.7
**193**	t5684	1	0.0	0.0	87.8
**194**	t5685	1	0.0	0.0	87.8
**195**	t5686	1	0.0	0.0	87.8
**196**	t5687	1	0.0	0.0	87.9
**197**	t5688	1	0.0	0.0	87.9
**198**	t5689	4	0.1	0.1	88.0
**199**	t5690	7	0.2	0.2	88.2
**200**	t571	25	0.8	0.8	89.0
**201**	t5721	3	0.1	0.1	89.1
**202**	t5758	3	0.1	0.1	89.2
**203**	t5759	10	0.3	0.3	89.5
**204**	t5760	4	0.1	0.1	89.7
**205**	t5761	1	0.0	0.0	89.7
**206**	t5775	1	0.0	0.0	89.7
**207**	t589	14	0.4	0.4	90.2
**208**	t5894	1	0.0	0.0	90.2
**209**	t591	11	0.3	0.3	90.5
**210**	t605	12	0.4	0.4	90.9
**211**	t617	6	0.2	0.2	91.1
**212**	t6172	1	0.0	0.0	91.1
**213**	t6191	1	0.0	0.0	91.2

**214**	t6192	1	0.0	0.0	91.2
**215**	t6193	9	0.3	0.3	91.5
**216**	t6194	8	0.3	0.3	91.7
**217**	t6195	5	0.2	0.2	91.9
**218**	t630	16	0.5	0.5	92.4
**219**	t6372	20	0.6	0.6	93.0
**220**	t6373	1	0.0	0.0	93.1
**221**	t6374	7	0.2	0.2	93.3
**222**	t6375	1	0.0	0.0	93.3
**223**	t6376	1	0.0	0.0	93.3
**224**	t645	3	0.1	0.1	93.4
**225**	t647	5	0.2	0.2	93.6
**226**	t676	10	0.3	0.3	93.9
**227**	t6762	2	0.1	0.1	94.0
**228**	t6763	1	0.0	0.0	94.0
**229**	t681	1	0.0	0.0	94.0
**230**	t686	1	0.0	0.0	94.1
**231**	t688	2	0.1	0.1	94.1
**232**	t693	5	0.2	0.2	94.3
**233**	t701	14	0.4	0.4	94.7
**234**	t7064	3	0.1	0.1	94.8
**235**	t7065	2	0.1	0.1	94.9
**236**	t7066	8	0.3	0.3	95.1
**237**	t7067	23	0.7	0.7	95.8
**238**	t712	4	0.1	0.1	96.0
**239**	t7170	1	0.0	0.0	96.0
**240**	t7267	1	0.0	0.0	96.0
**241**	t7271	2	0.1	0.1	96.1
**242**	t7272	1	0.0	0.0	96.1
**243**	t728	12	0.4	0.4	96.5
**244**	t746	1	0.0	0.0	96.5
**245**	t774	25	0.8	0.8	97.3
**246**	t779	1	0.0	0.0	97.4
**247**	t790	8	0.3	0.3	97.6
**248**	t796	1	0.0	0.0	97.6
**249**	t8027	1	0.0	0.0	97.7
**250**	t803	1	0.0	0.0	97.7
**251**	t837	4	0.1	0.1	97.8
**252**	t840	8	0.3	0.3	98.1
**253**	t884	5	0.2	0.2	98.2
**254**	t891	8	0.3	0.3	98.5
**255**	t909	1	0.0	0.0	98.5
**256**	t930	16	0.5	0.5	99.0
**257**	t950	3	0.1	0.1	99.1
**258**	t9883	1	0.0	0.0	99.2
**259**	t9884	10	0.3	0.3	99.5
**260**	t9886	7	0.2	0.2	99.7
**261**	t9887	1	0.0	0.0	99.7
**262**	t9888	1	0.0	0.0	99.7

**263**	t9889	1	0.0	0.0	99.8
**264**	t9894	5	0.2	0.2	99.9
**265**	t9896	1	0.0	0.0	100.0
**266**	t9897	1	0.0	0.0	100.0
	Total	3180	100.0	100.0	

Table S2. Most prevalent *spa*-types among the 3180 isolates.

Spa-Type	Number of Isolates	Prevalence
**t084**	182	7.2%
**t012**	111	4.4%
**t091**	110	4.3%
**t008**	91	3.6%
**t015**	83	3.3%
**t002**	63	2.5%

Table S3. IgG levels against *S. aureus* antigens in patients vs. healthy controls and estimated effect on FEV_1%.

Antigen	Mean IgG Level All Patients (± SE) [a]	Mean IgG Level Controls (± SE) [a]	*p* Value [b,c]	Estimated Effect on FEV1% [d]	*p* Value [c]
**CHIPS**	11492 (±220)	11019 (±349)	0.104	−0.00073	0.0185
**ClfA**	5168 (±237)	4302 (±436)	0.081	0.000514	0.1104
**ClfB**	4587 (±222)	4092 (±338)	0.3809	0.002079	<0.0001
**ETA**	3999 (±447)	2043 (±424)	0.1180	−0.00061	0.0033
**ETB**	613 (±113)	320 (±94)	0.0032	−0.00700	<0.0001
**FnbpA**	2922 (±201)	2534 (±300)	0.6167	0.000222	0.6310
**FnbpB**	967 (±79)	1327 (±215)	0.1306	−0.00199	0.1150
**HlgB**	13621 (±174)	9878 (±407)	<0.0001	−0.00109	0.0088
**LukF**	4079 (±133)	2718 (±243)	<0.0001	−0.00305	<0.0001
**LukS**	14097 (±133)	7134 (±524)	<0.0001	−0.00169	<0.0001
**SasG**	569 (±70)	636 (±132)	0.1847	0.001484	0.1590
**SdrD**	1084 (±80)	694 (±76)	0.0507	−0.00145	0.1950
**SdrE**	3139 (±201)	1992 (±221)	0.0253	−0.00179	<0.0001
**SEA**	3835 (±320)	3613 (±425)	0.2544	−0.00007	0.7599
**SEC**	7390 (±441)	8714 (±809)	0.1350	0.000269	0.1366
**SED**	1106 (±118)	1292 (±268)	0.0498	0.001490	0.0377
**SEE**	1333 (±183)	879 (±136)	0.8113	−0.00057	0.2779
**SEG**	2047 (±184)	1225 (±270)	0.0544	−0.00108	0.0264
**SEH**	2290 (±291)	2174 (±359)	0.0113	−0.00047	0.0986
**TSST1**	7981 (±487)	9076 (±646)	0.499	0.000148	0.3554

[a] Significant difference of results between 182 patients and 53 healthy controls; [b] *p* values of difference between patient and controls groups (Mann–Whitney U test); [c] adjusted *p*-values (Bonferroni correction); and [d] IgG levels are modelled as continuous factors. Estimated effects are therefore interpreted as mean change in FEV1% predicted.

Table S4 Primers for single and multiplex PCRs.

Virulence Gene	Primer	Sequence (5′–3′)	Reference
chp	*chp* forward	TTTACTTTTGAACCGTTTCCTAC	[1]
	chp reverse	CGTCCTGAATTCTTAGTATGCATATTCATTAG	
clf A	*clfA* forward	ATGGGACAACGAAGTAGCA	[2]
	clfA reverse	GCTTCATCTTCAGAACCTG	
clf B	*clfB* forward	GTTATGGTGGTGGAAGTGCTG	[2]
	clfB reverse	CGCTCTTATCTCCTGTTTCTGG	
fnb A	*fnbAB* forward	TAGGAACTGAAAATGGTCAC	[2]
	fnbA reverse	GAAGCAATCAGAAAACACTC	
fnb B	*fnbAB* forward	TAGGAACTGAAAATGGTCAC	[2]
	fnbB reverse	GAGTATGTAATTATTTCTTGG	
sdr C	*sdrC* forward	ACGACTATTAAACCAAGAAC	[3]
	sdrC reverse	GTACTTGAAATAAGCGGTTG	
sdr D	*sdrD* forward	GGAAATAAAGTTGAAGTTTC	[3]
	sdrD reverse	ACTTTGTCATCAACTGTAAT	
sdr E	*sdrE* forward	CAGTAAATGTGTCAAAAGA	[3]
	sdrE reverse	TTGACTACCAGCTATATC	
cna	*cna* forward	AGTGGTTACTAATACTG	[3]
	cna reverse	CAGGATAGATTGGTTTA	
cap	*cap5* forward	GAAAGTGAACGATTAGTAGAA	[4]
	cap5 reverse	GTACGAAGCGTTTTGATAGTT	
	cap8 forward	GTGGGATTTTTGTAGCTTTT	
	cap8 reverse	CGCCTCGCTATATGAACTAT	
sas G	*sasG* forward	GGGAACTCAACAAGAGGCAG	[5]
	sasG reverse	CAGAACGAGCTTTTCTAACC	
sas H	*sasH* forward	GTGTAATGGGATTATGGCAAG	[5]
	sasH reverse	CGTTGCTGTGTGAGTTGG	
sea	*sea*-3 forward	CCTTTGGAAACGGTTAAAACG	[6]
	sea-4 reverse	TCTGAACCTTCCCATCAAAAAC	
seb	*seb*-1 forward	TCGCATCAAACTGACAAACG	[6]
	seb-4 reverse	GCAGGTACTCTATAAGTGCCTGC	
sec	*sec*-3 forward	CTCAAGAACTAGACATAAAAGCTAGG	[6]
	sec-4 reverse	TCAAAATCGGATTAACATTATCC	
sed	*sed*-3 forward	CTAGTTTGGTAATATCTCCTTTAAACG	[6]
	sed-4 reverse	TTAATGCTATATCTTATAGGGTAAACATC	
see	see-2 forward	TAACTTACCGTGGACCCTTC	[6]
	see-3 reverse	CAGTACCTATAGATAAAGTTAAAACAAGC	
eta	*eta*-3 forward	CTAGTGCATTTGTTATTCAAGACG	[6]
	eta-4 reverse	TGCATTGACACCATAGTACTTATTC	
etb	*etb*-3 forward	ACGGCTATATACATTCAATTCAATG	[6]
	etb-4 reverse	AAAGTTATTCATTTAATGCACTGTCTC	
tst	*tst*-3 foward	AAGCCCTTTGTTGCTTGCG	[6]
	tst-6 reverse	ATCGAACTTTGGCCCATACTTT	
seg	*seg*-1 forward	AATGCTCAACCCGATCCTA	[7]
	seg-4 reverse	CTTCCTTCAACAGGTGGAGAC	

seh	*seh*-1 forward	TTAGAAATCAAGGTGATAGTGGC	[7]
	seh-2 reverse	TTTTGAATACCATCTACCCAAAC	
sei	*sei*-1 forward	GCCACTTTATCAGGACAATACTT	[7]
	sei-2 reverse	AAAACTTACAGGCAGTCCATCTC	
sej	*sej*-1 forward	CTCCCTGACGTTAACACTACTAATAA	[7]
	sej-2 reverse	TTGTCTGGATATTGACCTATAACATT	
agr	*agr*SA-KON1 forward	ATGCACATGGTGCACATGC	[8]
	*agr*SA1-2 reverse	GTCACAAGTACTATAAGCTGCGAT	
	*agr*SA2-2 reverse	TATTACTAATTGAAAAGTGCCATAGC	
	*agr*SA3-2 reverse	GTAATGTAATAGCTTGTATAATAATACCCAG	
	*agr*SA4-2 reverse	CGATAATGCCGTAATACCCG	
pvl	*pvl*-1 forward	ATCATTAGGTAAAATGTCTGGACATGATCCA	[9]
	pvl-2 reverse	GCATCAASTGTATTGGATAGCAAAAGC	
hlg	*hlg*-1 forward	GCCAATCCGTTATTAGAAAATGC	[9]
	hlg-2 reverse	CCATAGAAGTAGCAACGGAT	
eap	*eap*-CON1 forward	TACTAACGAAGCATCTGCC	[10]
	eap-CON2 reverse	TTAAATCGATATCACTAATACCTC	
emp	*emp*-1 forward	AATAATCGCGTGAATGTAG	[11]
	emp-2 reverse	CGTAGTAATGAAGTGGTGGT	

References

1. van Wamel, W.J.B.; Rooijakkers, S.H.M.; Ruyken, M.; van Kessel, K.P.M.; van Strijp, J.A.G. The Innate Immune Modulators Staphylococcal Complement Inhibitor and Chemotaxis Inhibitory Protein of Staphylococcus aureus Are Located on -Hemolysin-Converting Bacteriophages. *J. Bacteriol.* **2006**, *188*, 1310–1315.
2. Gomes, a R.; Vinga, S.; Zavolan, M.; Lencastre, H. De Analysis of the Genetic Variability of Virulence-Related Loci in Epidemic Clones of Methicillin-Resistant Staphylococcus aureus. *Antimicrob. Agents Chemother.* **2005**, *49*, 366–379.
3. Peacock, S.J.; Moore, C.E.; Justice, A.; Kantzanou, M.; Story, L.; Mackie, K.; O'Neill, G.; Day, N.P.J. Virulent Combinations of Adhesin and Toxin Genes in Natural Populations of Staphylococcus aureus. *Infect. Immun.* **2002**, *70*, 4987–4996.
4. Goerke, C.; Esser, S.; Kümmel, M.; Wolz, C. Staphylococcus aureus strain designation by agr and cap polymorphism typing and delineation of agr diversification by sequence analysis. *Int. J. Med. Microbiol.* **2005**, *295*, 67–75.
5. Roche, F.M.; Massey, R.; Peacock, S.J.; Day, N.P.J.; Visai, L.; Speziale, P.; Lam, A.; Pallen, M.; Foster, T.J. Characterization of novel LPXTG-containing proteins of Staphylococcus aureus identified from genome sequences. *Microbiology* **2003**, *149*, 643–654.
6. Becker, K.; Roth, R.; Peters, G. Rapid and specific detection of toxigenic Staphylococcus aureus: Use of two multiplex PCR enzyme immunoassays for amplification and hybridization of staphylococcal enterotoxin genes, exfoliative toxin genes, and toxic shock syndrome toxin 1 gene. *J. Clin. Microbiol.* **1998**, *36*, 2548–2553.
7. Becker, K.; Friedrich, A.W.; Lubritz, G.; Weilert, M.; Peters, G.; Eiff, C. Von Prevalence of Genes Encoding Pyrogenic Toxin Superantigens and Exfoliative Toxins among Strains of. *Microbiology* **2003**, *41*, 1434–1439.
8. Lina, G.; Boutite, F.; Tristan, A.; Bes, M.; Etienne, J.; Vandenesch, F. Bacterial competition for human nasal cavity colonization: Role of Staphylococcal agr alleles. *Appl. Environ. Microbiol.* **2003**, *69*, 18–23.
9. Von Eiff, C.; Friedrich, A.W.; Peters, G.; Becker, K. Prevalence of genes encoding for members of the staphylococcal leukotoxin family among clinical isolates of Staphylococcus aureus. *Diagn. Microbiol. Infect. Dis.* **2004**, *49*, 157–162.
10. Hussain, M.; Von Eiff, C.; Sinha, B.; Joost, I.; Herrmann, M.; Peters, G.; Becker, K. eap gene as novel target for specific identification of Staphylococcus aureus. *J. Clin. Microbiol.* **2008**, *46*, 470–476.
11. Hussain, M.; Becker, K.; Von Eiff, C.; Schrenzel, J.; Peters, G.; Herrmann, M. Identification and characterization of

a novel 38.5-Kilodalton cell surface protein of Staphylococcus aureus with extended-spectrum binding activity for extracellular matrix and plasma proteins. *J. Bacteriol.* **2001**, *183*, 6778–6786.
